# THE SEARCH STUDY: A QUALITATIVE INQUIRY INTO DIVERSE STAKEHOLDER PERSPECTIVES ON HEALTHY AGING ACROSS CHICAGOLAND

**DOI:** 10.1093/geroni/igz038.3484

**Published:** 2019-11-08

**Authors:** Mushira Khan, Karen Graham, Tarisha Washington, Abigail Kim, Raj C Shah, Crystal M Glover

**Affiliations:** 1 Rush Alzheimer's Disease Center, Illinois, United States; 2 Rush Alzheimer's Disease Center, Chicago, Illinois, United States; 3 Rush University, Chicago, Illinois, United States

## Abstract

Older adults face increased risk of chronic diseases of aging such as Alzheimer’s dementia and other adverse age-related outcomes. However, the conceptualization of healthy aging and how age-related issues are addressed in community-based structures, particularly among racial and ethnic minorities, remain poorly understood, especially from the Stakeholder perspective. Stakeholders, such as faith leaders and members of community-based organizations, engage in regular negotiations to advance health equity in their communities by partnering and collaborating with older adults and their families as well as other local and federal organizations. The Stakeholder Engagement in Aging Research and Community Health (SEARCH) Study employs multiple research methods to illuminate Stakeholders’ perspectives on barriers and facilitators to healthy aging in diverse communities. This presentation highlights findings from in-depth, qualitative interviews with Stakeholders (N=37) serving African American, Latinx, and South Asian older adults. Emergent themes suggest that systemic racism, stigmatization, limited health literacy, and cultural beliefs serve as barriers to healthy aging across groups. Within groups, Stakeholders report precarious immigration status and fragile and fragmented life situations as barriers among Latinx older adults, while acculturative stress presents a challenge to healthy aging in South Asian older adults. Food insecurity and neighborhood factors such as exposure to violence and socio-economic disadvantages act as barriers among African American older adults. Conversely, religious faith and spirituality, familial support, and culturally-congruent care serve as facilitators across groups. The findings from this study underscore the continued need for intersectional, inclusive, and culturally-informed approaches to supporting healthy aging within diverse communities.

